# Simulated seasonal diets alter yak rumen microbiota structure and metabolic function

**DOI:** 10.3389/fmicb.2022.1006285

**Published:** 2022-09-23

**Authors:** Xugang Yang, Xueni Fan, Hui Jiang, Qiang Zhang, Basangwangdui, Qunying Zhang, Siqi Dang, Ruijun Long, Xiaodan Huang

**Affiliations:** ^1^School of Public Health, Lanzhou University, Lanzhou, China; ^2^School of Ecology, Lanzhou University, Lanzhou, China; ^3^State Key Laboratory of Barley and Yak Germplasm Resources and Genetic Improvement, Tibet Academy of Agricultural and Animal Husbandry Sciences (TAA AS), Lhasa, China

**Keywords:** yak, volatile fatty acid, season, nutrient simulation, rumen microbiota, metagenomics

## Abstract

Yak is the only ruminant on the Qinghai-Tibetan Plateau that grazes year-round. Although previous research has shown that yak rumen microbiota fluctuates in robust patterns with seasonal foraging, it remains unclear whether these dynamic shifts are driven by changes in environment or nutrient availability. The study examines the response of yak rumen microbiota (bacteria, fungi, and archaea) to simulated seasonal diets, excluding the contribution of environmental factors. A total of 18 adult male yaks were randomly divided into three groups, including a nutrition stress group (NSG, simulating winter pasture), a grazing simulation group (GSG, simulating warm season pasture), and a supplementation group (SG, simulating winter pasture supplemented with feed concentrates). Volatile fatty acids (VFAs) profiling showed that ruminal acetate, propionate and total VFA contents were significantly higher (*p* < 0.05) in GSG rumen. Metagenomic analysis showed that *Bacteroidetes* (53.9%) and *Firmicutes* (37.1%) were the dominant bacterial phyla in yak rumen across dietary treatments. In GSG samples, *Actinobacteriota*, *Succinivibrionaceae_UCG-002*, and *Ruminococcus albus* were the most abundant, while *Bacteroides* was significantly more abundant in NSG samples (*p* < 0.05) than that in GSG. The known fiber-degrading fungus, *Neocallimastix,* was significantly more abundant in NSG and SG samples, while *Cyllamyces* were more prevalent in NSG rumen than in the SG rumen. These findings imply that a diverse consortium of microbes may cooperate in response to fluctuating nutrient availability, with depletion of known rumen taxa under nutrient deficiency. Archaeal community composition showed less variation between treatments than bacterial and fungal communities. Additionally, *Orpinomyces* was significantly positively correlated with acetate levels, both of which are prevalent in GSG compared with other groups. Correlation analysis between microbial taxa and VFA production or between specific rumen microbes further illustrated a collective response to nutrient availability by gut microbiota and rumen VFA metabolism. PICRUSt and FUNGuild functional prediction analysis indicated fluctuation response of the function of microbial communities among groups. These results provide a framework for understanding how microbiota participate in seasonal adaptations to forage availability in high-altitude ruminants, and form a basis for future development of probiotic supplements to enhance nutrient utilization in livestock.

## Introduction

The Qinghai-Tibetan Plateau is the highest and largest plateau in the world, and is utilized for ruminant grazing year-round (Long et al., 1999), besides playing vital ecological roles as a watershed and ruminant habitat (Yu et al., 2012). Yak (*Bos grunniens*), referred to in Chinese as the “boat of the plateau,” is the only large mammal endemic to the Qinghai-Tibet Plateau, and yak husbandry is tightly interwoven in the lifestyle of the Tibetan people, providing indispensable goods, such as dairy, meat, textiles, and fuel (Long et al., 1999). As a result of their prolonged development and adaptation to survival on the plateau, yaks can withstand extreme cold, high ultraviolet radiation, hypoxia, and seasonally low nutrient availability (Qiu et al., 2012; Zhang et al., 2016). This adaptation to harsh environments raises numerous questions about the function and dynamics of their resident rumen microbiota, and especially the role of rumen microbiota in nutrient utilization efficiency under wide variation of pasture conditions.

Rumen microbiota perform integral functions in nutrient utilization, metabolism, immune function, animal health, and even host developmental processes (Heijtza et al., 2011; Sonnenburg and Backhed, 2016; Islam et al., 2019). Dietary and feeding regimens can also reportedly affect the composition of bacterial and archaeal communities in yak rumen (Xue et al., 2017; Zhou et al., 2017; Liu et al., 2019), and their microbial symbionts have been shown to ferment plant proteins and polysaccharides (Deusch et al., 2017), and provide the host with essential nutrients, volatile fatty acids (VFAs), and microbial proteins obtained from relatively recalcitrant plant fibers (Ishaq and Wright, 2012). Previous studies in yak have found that rumen microbiota composition is not only related to feed efficiency (Henderson et al., 2015), but also subject to co-evolution with the host genome as part of the adaptive response to extreme natural environments (Zhang et al., 2016). Guo et al. (2021) and Huang et al. (2022) demonstrated that seasonal constraints on forage availability led to restructuring of the yak rumen microbiota, enriching for fiber-degrading microbiota in the winter season when low-nutrient, high-fiber plant residue comprises the majority of the food supply. However, the contribution of rumen fungi, which are well-established microbial fiber degraders, has been largely overlooked in previous investigations of seasonal nutrient variation. Moreover, environmental factors can also impact rumen microbiota composition and diversity, and therefore, experiments simulating different nutrient availability conditions can promote a more robust comprehension of yak rumen community adaptation to seasonal changes in forage conditions, and identify functionally relevant microorganisms that are enriched under different conditions.

In the present study, we investigated the influence of seasonal changes in nutrient availability on yak rumen microbiota (including bacteria, fungi, and archaea) by simulating grazing conditions in winter pasture, warm season pasture, and winter pasture with supplementation. To this end, high throughput metagenomic rRNA gene sequencing was used to characterize rumen community composition and diversity under different grazing conditions. Then, to test the hypothesis that specific microbes are enriched under different grazing conditions, leading to differences in rumen fermentation, we characterized the volatile fatty acid (VFA) profiles of all rumen samples and conducted correlation analysis between microbial taxa (ASVs) and different VFAs. This study extends our understanding of the role of rumen microbes in metabolic adaptation to extreme seasonal changes in forage availability.

## Materials and methods

### Animal selection and experimental design

Procedures of the current study were approved by the Animal Welfare and Ethics Committee of Lanzhou University (Approval Reference Number: EAF2021033). The study was conducted in August 2021, at the Yak Breeding Cooperative in Nimu County, Lhasa City (29°36′N, 90°6′E 4,230 m a.s.l.).The experiment selected 18 male yaks (350 ± 26 kg live weight, aged 4–5 years) and randomly divided into three different diet groups (shown in Table 1). The experiment was carried out over a period of 35 days, of which the first 14 days were for adaptation and the next 21 days for experiment and sampling. The test was divided into three groups: Grazing simulation group (GSG), Nutrition stress group (NSG) and Supplementation group (SG) according to the nutrient intake and average daily gain of yaks grazing in different seasons (Xue et al., 2005, 2007), the Feeding Standards of Beef Cattle (NY-T815-2004) and the nutrient changes of forage grass in different seasons on the Qinghai-Tibet Plateau (Long et al., 1999; Guo et al., 2021). In which, Nutrient stress group (NSG) was considered as fed deficiency group according to the nutrient level of local winter pasture; Grazing simulation group (GSG) simulated the nutrient intake of yak while grazing in warm-season pasture; Supplementation group (SG) simulated the nutrient intake of yak while grazing in the winter pasture with supplementation. The yaks were fed twice a day at 8:30 and 16:30 h at 2% BW on a dry matter basal diet and allowed *ad libitum* access to water. The remaining feed was collected and weighed before morning feeding to calculate the actual feed intake.

**Table 1:** Diet composition of three treatment groups.

Ingredients	Proportion (% of DM)		
	NSG	GSG	SG
Concentrate	0	10.8	27.7
Corn silage	17.1	7.0	0
Oaten hay	58.9	59.6	48.4
Alfalfa hay	21	19.6	20.9
CaCO_3_	0.5	0.5	0.5
NaHCO₃	0.5	0.5	0.5
NaCl	1	1	1
Premix	1	1	1
Total	100	100	100
**Nutrition contents of diet**			
CP, % of DM	7.72	10.11	13.93
*CF*, % of DM	23.15	20.89	19.68
EE, % of DM	2.78	3.09	3.13
Ca, % of DM	0.36	0.39	0.38
P, % of DM	0.25	0.26	0.31
DE (MJ/kg DM)	9.44	11.07	12.30

1. Premix contained per kilogram: 41 mg Fe, 1.2 mg I, 0.5 mg Co, 98 mg Mn,16 mg Ca, 5 mg P, 0.9 mg Se, 187 mg Zn, 36 mg Cu, VA 1500 IU, VD 550 IU, VE 10 IU.

2. Concentrate contained per kilogram: 610 g maize grain, 70 g soy bean meal, 200 g wheat bran, 120 g wheat bran.

3. DMI: NSG 4.5 kg/day; GSG 5.3 kg/day; sg 6.3 kg/day.

4. DM, dry matter; DMI, dry matter intake; CP, crude protein; CF, crude fiber; EE, ether extract; DE, digestive energy.

### Sample collection

On the last day (day 35) of the experiment, a stomach tube (Kelibo Co. Ltd., Wuhan, China) was used to collect rumen fluid from each yak before morning feeding. The initial 50 ml of fluid were discarded to avoid contamination by saliva. Then, 40 ml of rumen fluid was taken and stored in a sterile enzyme-free cryopreservation tube with liquid nitrogen freezer and transferred immediately to the laboratory and stored in a –80°C refrigerator. The collected samples were mainly used for the determination of volatile fatty acids (VFAs) and microbial community analysis.

### Determination of VFAs

Volatile fatty acids (VFAs) were determined by modified method from Erwin et al. (1961). The rumen fluid was firstly thawed at 4°C and centrifuged at 3850×*g* at 4°C for 15 min, metaphosphoric acid solution(0.2 ml) containing 2-ethyl butyric acid as internal standard was added to supernatant(1 ml). After mixing well and standing for 30 min at 4°C. The mixed solution was centrifuged at 3850×*g* at 4°C for 15 min, and the supernatant was collected and filtered (0.22 μm) for VFAs determination using a gas chromatograph (GC, SP-3420A; Beijing Beifen-Ruili Analytical Instrument Co., Ltd., Beijing, China) with an AT-FFAP type capillary column (30 m × 0.32 mm × 0.5 μm) and a flame ionization detector, with the temperature regime as: 90°C for 1 min, increased to 120°C at 10°C/min for 1 min, then increased from 120 to 150°C at 10°C/min, and maintained at 150°C for 3 min. The injection hole temperature was 250°C, and the auxiliary chamber temperature was 250°C.

### Diet and chemical analysis

The collected feed samples were dried in a forced-air oven at 65°C for 48 h. After crushed and then passed through a 1 mm sieve, feed samples were weighed and dried at 105°C to a constant weight to thus subjected to dry matter (DM) and crude protein (CP) determination by the Kjeldahl method (Thiex et al., 2012), while neutral detergent fiber (NDF) and acid detergent fiber (ADF) were measured by an automatic fiber analyzer (ANKOM 2000i, ANKOM Technology, Macedon, NY, USA) (Vansoest et al., 1991). Determination of crude ash in diet was carried out according to the method described by Thiex et al. (2012) and ether extract (EE) was measured according to the method described by Cunniff and Association of Official Analytical Chemists (1995).

### DNA extraction, sequencing and data analysis

Rumen fluid stored at −80°C was subjected to genomic DNA extraction using TIANamp Stool DNA Kit (Tiangen Biotech Co., Ltd., Beijing, China). DNA spectrophotometer (ND-1000; NanoDrop, Wilmington, DE, United States) was used for DNA yield and purity screening. Normalized genomic DNA was applied to perform barcode PCR with a set of primers (338\u00B0F and 806R for bacteria; ITS1-1F-F-ITS1-1F-R for Fungi and 1106F-1378R for Archaea). Phusion® High-Fidelity PCR Master Mix (New England Biolabs) was used in the PCR reactions, cycling conditions were as follows: 1. denaturation step at 98°C, 1 min; 2. 30 cycles at 98°C for 10s, 50°C for 30 s, 72°C for 30 s; and 3. a final 5 min extension at 72°C. The PCR products were thus subjected to agarose gel electrophoresis for separation and visualization. The final products were sequenced on an Illumina NovaSeq platform and 250 bp paired-end reads were generated.

Paired-end reads were assigned to each sample by barcodes identification. After cutting off the barcodes and primer sequences, Paired-end reads were merged and spliced by FLASH v 1.2.11 (Magoč and Salzberg, 2011). Quality filtering was performed using the fastp (Version 0.20.0), after getting rid of noise and chimeric reads by QIIME2 (Version QIIME2-202006) DADA2 and Vsearch (Version 2.15.0; Li et al., 2020), sequences are then clustered into ASVs (Amplicon Sequence Variants) (default: DADA2) based on the database. The database used for the taxonomic assignment comes from Silva database^1^ for 16S and Unite database^2^ for ITS (Haas et al., 2011).

Alpha and beta diversity were calculated from different indices in QIIME2 (Version QIIME2-202006). Principal Coordinate Analysis (PCoA) was used to obtain principal coordinates and visualize differences of rumen samples in complex multi-dimensional data. The results were plotted using ade4 and ggplot2 packages in R. LEfSe analysis was performed through the Huttenhower Lab Galaxy Server^3^. The PICRUSt software (Version 2.1.2-b) was used to predict the function of bacteria and archaea (Douglas et al., 2020). The FUNGuild software was conducted to assign ecological functions to each ASVs (Nguyen et al., 2016). The original 16S rRNA/ITS data were available at the NCBI SRA database with accession number PRJNA863131^4^.

### Statistical analyses

The association between rumen microbiota and VFA through Spearman’s correlation analysis. One-way ANOVA by SAS version 9.2 was used to compare rumen VFAs. Non-parametric factorial Kruskal–Wallis sum-rank tests were performed to test for differences among groups at the bacterial phylum and genus levels, and Dunn’s test was performed to separate means where significance was found. Tukey-adjusted *p* values were used to separate means and statistical significance was accepted at *p* < 0.05.

## Results

### Rumen VFAs increase under warm season grazing simulation (GSG)

In order to better understand the differences in microbial-associated metabolic processes in yak rumen under varying nutrient availability, volatile fatty acid (VFA) composition was determined in yak rumen fluid of animals treated with simulated nutrient deficiency (NSG), simulated warm season grazing (GSG), or supplemented cold season grazing (SG) (*n* = 6 animals per treatment; Table 2). The results suggested that diet (i.e., nutrient levels) affected the amounts of rumen acetate, propionate, and total VFAs, which were significantly higher (*p* < 0.05) in GSG than in NSG or SG, while the concentrations of rumen butyrate and valerate, as well as the acetate: propionate ratio showed no difference among the three experimental groups. These results suggested that metabolic processes in yak rumen indeed varied with nutrient intake, leading us to hypothesize that rumen microbiota contributed to this process.

**Table 2:** Volatile fatty acids (VFAs) composition affected by different nutrient simulations.

Items	NCG	SG	GSG	SEM	*p*-value
VFAs, mmol/L					
Acetate	24.82^a^	24.67^a^	31.93^b^	1.24	<0.01
Propionate	5.50^a^	5.56^a^	6.75^b^	0.23	0.02
Isobutyrate	0.38	0.39	0.41	0.01	0.49
Butyrate	2.05	1.97	2.56	0.14	0.15
Isovalerate	0.52	0.59	0.56	0.02	0.52
Valerate	0.21	0.25	0.256	0.01	0.14
A/P	4.52	4.43	4.73	0.08	0.24
TVFA, mmol/L	33.46^a^	33.43^a^	42.48^b^	1.60	0.01

TVFAs, total volatile fatty acids; A/P, acetate/propionate.

^a,b^Means within a row followed by different lower case letters differ significantly from each other (p < 0.05).

### Rumen microbiota

#### Bacteria diversity and community shift associated with nutrient fluctuation

To identify differences in yak rumen microbiota associated with nutrient fluctuation, metagenomic sequencing of the 16S rRNA gene was conducted in rumen samples of yaks fed under NSG, GSG and SG conditions. In total, 1,959,743 cleaned reads were obtained from 18 samples after quality control and filtering, with an average of 69,564 valid sequences per sample. Denoising with DADA 2A revealed that 3,341 ASVs were present in the three groups combined, among which 2068 ASVs were detected in all three groups, while 245 were unique to NSG, 192 were exclusively detected in SG, and 265 were only present in the GSG rumen (Supplementary Figure S1A).

**Figure 1:**
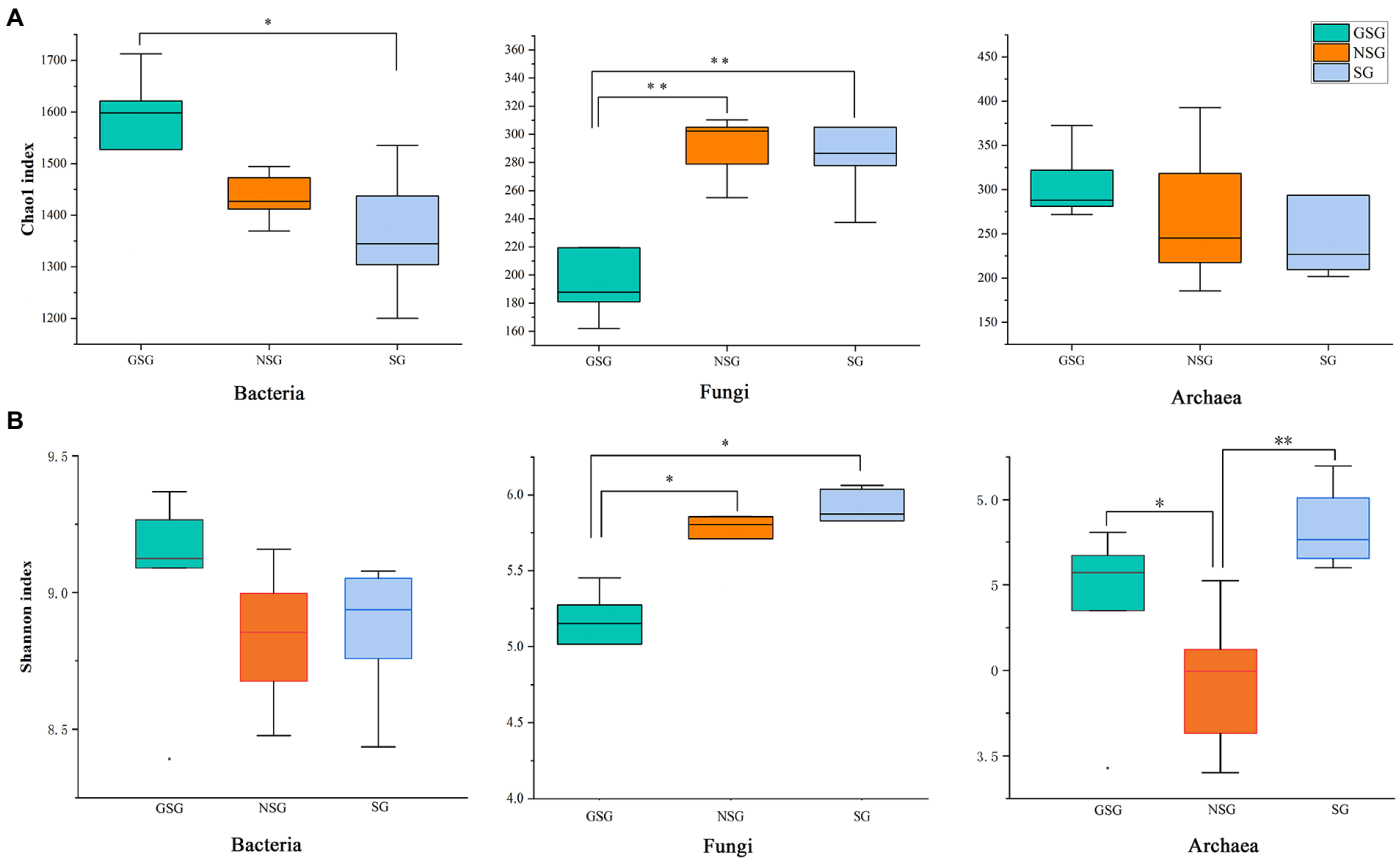
Differences in Yak rumen microbial diversity and richness between the GSG, NSG, and SG. Diversity was estimated by Shannon index. Richness estimated by the Chao1 value. **(A)** Chao 1 index. **(B)** Shannon index. Asterisks indicate significant difference between the Concentrate Group and the Forage Group (*p ≤ 0.05; **p ≤ 0.01).

**Figure 2:**
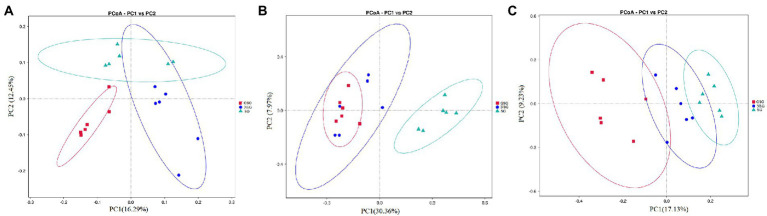
Principal coordinate analysis (PCoA) of rumen microbial communities. **(A)** Bacteria. **(B)** Fungi. **(C)** Archaea.

**Figure 3:**
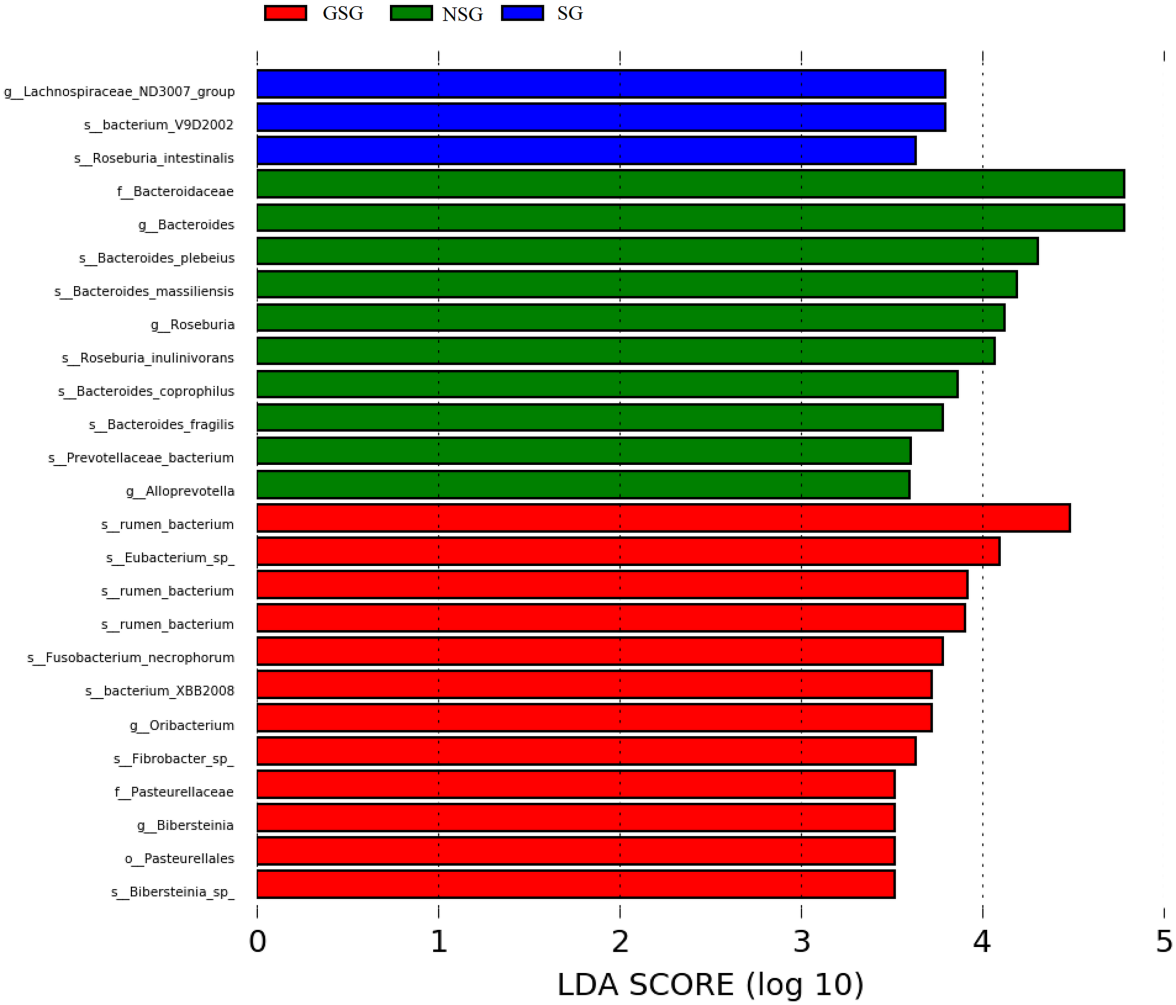
Linear discriminant analysis (LDA) of rumen bacteria. (LDA cutoff of +/−2.0).

Analysis of the alpha diversity of rumen bacteria indicated that species richness (Chao 1 index) was significantly higher in the GSG rumen (1611) than in the SG rumen (1360) (*p* < 0.05), while NSG had intermediate richness between the other treatments (Figure 1A). Shannon diversity indices were not significantly different among groups (*p* > 0.05) (Figure 1B). Principal coordinates analysis (PCoA) based on an unweighted UniFrac distance matrix to visualize overall structural changes in rumen bacterial communities showed obvious separation of the three groups (Figure 2A), with GSG rumen bacteria exhibiting the tightest clustering. LEfSe analysis to identify indicator taxa under different nutrient conditions revealed 10 (*Bacteroides*, *Roseburia,* etc.), 12 (*Oribacterium*, *Pasteurellales,* etc.) and 3 (*Lachnospiraceae_ND3007_group*, *Roseburia_intestinalis,* etc.) bacterial taxa significantly associated with NSG, GSG and SG yak rumen, respectively (Figure 3).

**Figure 4:**
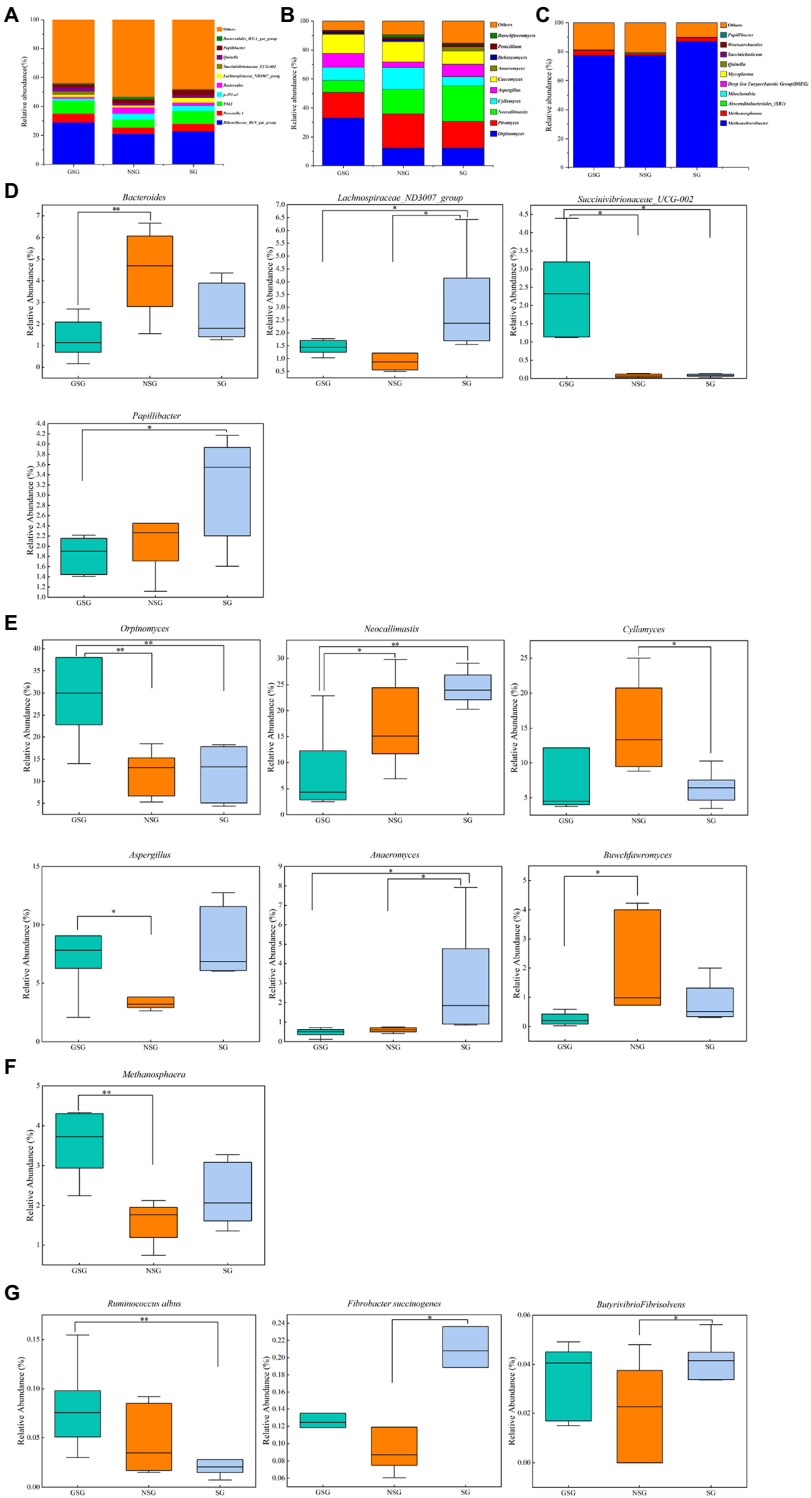
Classification of the rumen microbial composition at the genus level across the different nutrient simulations in different grazing patterns. **(A)** Bacteria. **(B)** Fungi. **(C)** Archaea. **(D)** Bacterial genera with significant changes under various nutrient simulations. **(E)** Fungal genera with significant changes under various nutrient simulations. **(F)** Archaeal genera with significant changes under various nutrient simulations. **(G)** Major fiber-degrading bacteria with significant changes under various nutrient simulations. Asterisks indicate significant difference between the three groups (*p ≤ 0.05; **p ≤ 0.01).

A total of 21 bacterial phyla and 336 bacterial genera were identified in the combined experimental samples. *Bacteroidetes* (53.97%) and *Firmicutes* (37.16%) were the dominant phyla in all three treatment groups (Supplementary Figure S2A), with *Proteobacteria* and *Actinobacteriota* (5.40% and 1.08%, respectively) comprising the next most abundant taxa. These four phyla with highest relative abundance accounted for >90% of all bacterial ASVs. At the genus level, the predominant genera included *Rikenellaceae_RC9_gut_group* (24.25%), *uncultured_bacterium_f_F082* (8.01%), and *Prevotella_1* (5.12%) in all samples (Figure 4A). Less prevalent but relatively abundant genera included *p-251-o5*, *Bacteroides*, *Papillibacter*, *Quinella,* and *Lachnospiraceae_ND3007_group,* respectively accounting for 2.81%, 2.71%, 2.41%, 2.36% and 1.97% of detected ASVs.

At the phylum level (Supplementary Figure S2D), in comparisons with the NSG yak rumen, the GSG rumen had a higher abundance of *Bacteroidetes* (57.2% vs. 50.4%) but lower abundance of *Firmicutes* (33.4% vs. 39.4%; *p* < 0.05). Yak rumen from GSG also had the highest abundance of *Actinobacteriota* among all the groups (2.1% vs. 0.5% and 0.5%; *p* < 0.05). At the genus level, the relative abundance of *Bacteroides* in NSG was higher than that in the GSG rumen (4.4% vs. 2.4%; *p* < 0.05), while *Lachnospiraceae_ND3007_group* was significantly enriched in SG (3.1% vs. 1.3% and 1.4%; *p* < 0.05) and *Succinivibrionaceae_UCG-002* was significantly higher in GSG rumen (2.2% vs. 0.06% and 0.08%; *p* < 0.05) compared with other groups (Figure 4D). In addition, the differences in nutrient availability significantly affected (*p* < 0.05) species level ASVs such as *Fibrobacter succinogenes*, *Butyrivibrio fibrisolvens,* and *Ruminococcus albus*. The relative abundance of *Fibrobacter succinogenes* and *Butyrivibrio fibrisolvens* in SG was significantly higher (0.2% vs. 0.1% and 0.04% vs. 0.02%; *p* < 0.05) than that in NSG, while GSG rumen had the higher abundance of *Ruminococcus albus* than that in SG group (0.08% vs. 0.02%, *p* < 0.05) (Figure 4G). These cumulative results suggested that the nutrient deficiency can inhibit proliferation of known rumen taxa, while selecting for nutrient stress-tolerant, fiber-degrading bacteria and fungi.

#### Higher rumen fungi diversity and significant taxa in NSG and SG rumen

Based on the observed differences in bacterial communities between treatments, metagenomic sequencing of the fungal ITS hypervariable region was conducted to examine changes in rumen fungi, which are well known to contribute to cellulose degradation in ruminants. In total, 92,749 cleaned reads were obtained for each sample after splicing and filtering, with a total of 1,441 ASVs detected in the three groups combined after denoising. Among these fungal ASVs, 213 were common to all three groups, while 395 were unique to NSG rumen, 383 were unique to SG, and 200 were exclusively detected in GSG (Supplementary Figure S1B).

Examination of fungal diversity (Shannon index) and species richness (Chao 1) in yak rumen indicated that both indexes were lower in GSG than in the NSG and SG rumen (*p* < 0.01) (Figures 1A,B). PCoA analysis showed no obvious distinction between rumen fungal communities of the NSG and SG rumen, whereas GSG was clearly separated from the other two groups (Figure 2B). Subsequent LEfSe analysis identified 7 (*Buwchfawromyces*, *Kazachstania,* etc.), 17 (*Eurotiales*, *Aspergillaceae,* etc.), and 26 (*Melanocarpus*, *Neocallimastix,* etc.) fungal taxonomic groups that were significant indicator taxa in the NSG, GSG, and SG rumen, respectively (Supplementary Figure S3A).

A total of 7 fungal phyla and 122 fungal genera were identified in the three groups. In particular, *Neocallimastigomycota* (81.29%) and *Ascomycota* (12.91%) were the most abundant phyla in all rumen samples (Supplementary Figure S2B), followed by *Basidiomycota*, *Mortierellomycota,* and *Mucoromycota* (0.76, 0.10 and 0.03%, respectively). At the genus level, predominant genera included *Piromyces* (20.13%), *Orpinomyces* (19.01%), and *Neocallimastix* (16.56%) in all samples. Less prevalent but significantly abundant genera included *Caecomyces*, *Cyllamyces*, *Aspergillus*, *Anaeromyces,* and *Penicillium,* accounting for 12.00, 10.26, 7.16, 1.37, and 1.28% of fungal ASVs, respectively (Figure 4B).

Examination of phylum-level community composition showed that *Mortierellomycota* in SG was significantly higher than in GSG (*p* < 0.05 Supplementary Figure S1E), whereas, at the genus level, the relative abundance of *Orpinomyces* was higher in the GSG rumen than in the NSG and SG rumen (33.0% vs. 12.0% and 11.9%; *p* < 0.05) and *Neocallimastix* was lower (8.2% vs. 17.2% and 24.4%; *p* < 0.05). Yak rumen in the SG rumen had the highest abundance of *Anaeromyces* among the three groups (3.0% vs. 0.6% and 0.5%, *p* < 0.05), while *Cyllamyces* was enriched in NSG compared to SG (15.1% vs. 6.5%, *p <* 0.05), and *Buwchfawromyces* was more abundant in NSG than in the GSG rumen (*p* < 0.05) (Figure 4E). In summary, these results indicated that nutrient utilization is a complex process which requires a diverse consortium of microbes working together, and thus builds a plasticity to allow fungi to response to different substrates.

#### Rumen archaeal community vary under different nutrient conditions

In addition to bacterial and fungal contribution to rumen metabolism, we hypothesized that rumen archaea may also vary under different nutrient conditions. To test this possibility, metagenomic sequencing of the archaeal was conducted in rumen of yaks from each treatment group, which resulted in 1,699,212 clean reads, after quality control and filtering, from all samples combined. On average, 83,546 valid sequences were obtained for each sample, which contained 1,997 total ASVs. There were 164 ASVs shared by all three groups, while much larger proportions were unique to each grazing simulation, including 545 ASVs exclusive to NSG rumen, 428 ASVs unique to SG rumen, and 481 ASVs only found in GSG rumen (Supplementary Figure S1C).

Comparison of rumen archaeal diversity (Shannon index) indicated that SG (4.8) had significantly higher diversity than in GSG (4.4) and NSG (3.9) (*p* < 0.05), although species richness (Chao 1) was not significantly different among the three groups (Figures 1A,B). PCoA analysis showed that archaeal communities clustered into distinct groups based on nutrient conditions, with little overlap (Figure 2C). LeFse analysis of indicator taxa for each nutrient condition group identified *Methanobrevibacter* in NSG, *Methanosphaera* and *Methanobrevibacter ruminantium* in GSG rumen, and *Methanobrevibacter millerae* in the SG rumen (Supplementary Figure S3B). Examination archaeal community composition identified four distinct phyla and six distinct genera that were present in all groups, including *Euryarchaeota,* which was the most abundant phylum in all samples, accounting for 84.19% of archaeal ASVs (Supplementary Figure S2C). Significant, but less prevalent phyla included *Halobacterota* (0.15%) and *Aenigmarchaeota* (0.02%). At the genus level, *Methanobrevibacter* (81.03%) and *Methanosphaera* (2.47%) were predominant across samples (Figure 4C). Further analysis by one-way ANOVA showed that the relative abundance of *Methanosphaera* in NSG was significantly lower than in GSG (*p* < 0.05), but did not differ from that in SG (Figure 4F). Collectively, these results found that sufficient nutrient conditions could enhance VFA production by enrich less hydrogen consumer-*Methanosphaera*.

#### Functional prediction of rumen bacteria, archaea, and fungi

A total of 69 enriched metabolic pathways were predicted based on identified bacteria and archaea amplicon sequences using the KEGG pathway database, and thus were mainly divided into three categories as metabolism, biosynthesis, and cellular processes (Supplementary Figures S4A,C). We found that the Calvin–Benson–Bassham cycle and 5-aminoimidazole ribonucleotide biosynthesis were significantly enriched in GSG when compared with that in NSG (*p* < 0.05). In addition, fungal function prediction using FUNGuild software showed that pathotrophs, symbiotrophs, and saprotrophs were the major components. Plant_Pathogen and Endophyte-Plant_Pathogen were significantly enriched in SG than that in NSG (*p* < 0.05), and Animal_Pathogen-Endophyte-Plant_Pathogen-Wood_Saprotroph was significantly more abundant in SG than that in GSG (*p* < 0.05). Moreover, the relative abundance of Plant_Pathogen-Soil_Saprotroph-Wood_Saprotroph was significantly higher in GSG than that in NSG (*p* < 0.05) (Supplementary Figure S4B).

#### Significant interactions between rumen microbes and volatile fatty acids

**Figure 5:**
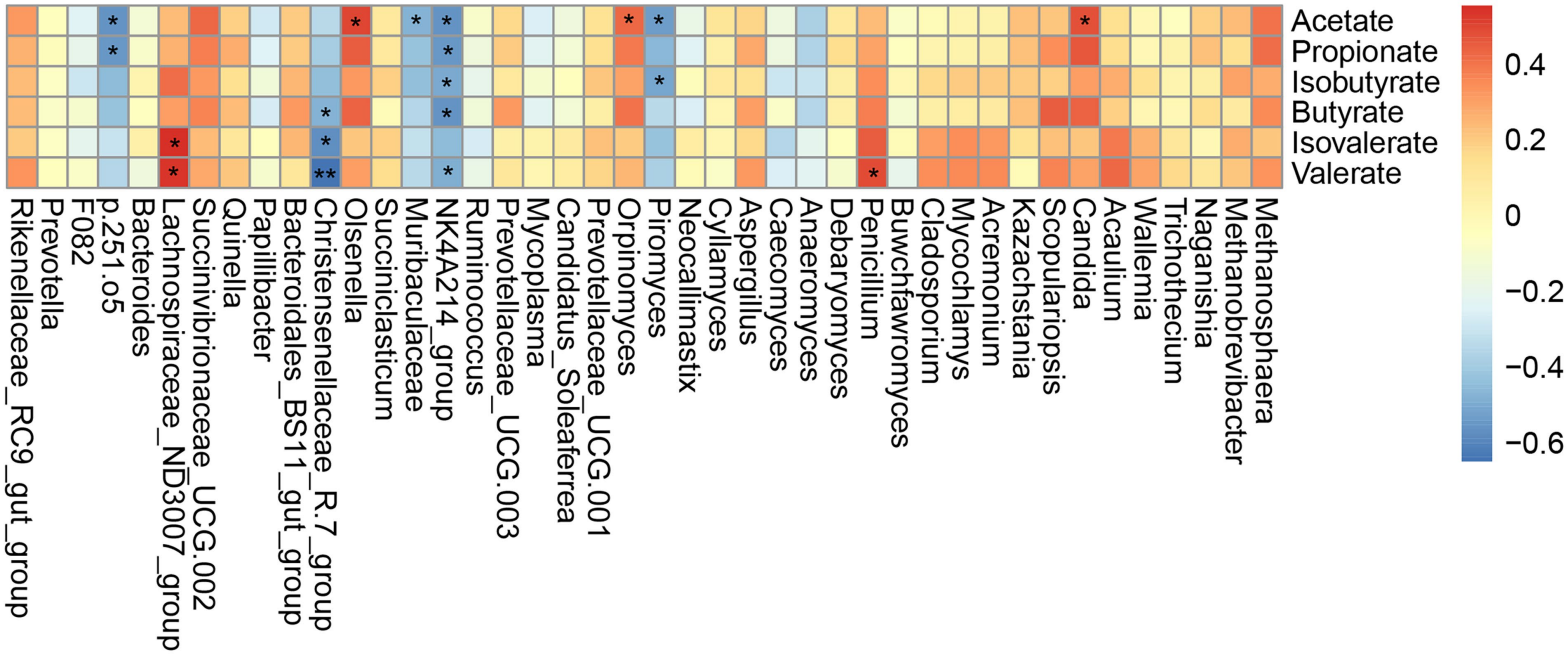
Correlations between the higher abundant genera of rumen microbial and VFAs. Each row in the graph represents a genus, each column represents a metabolite, and each lattice represents a Spearman’s correlation coefficient between a component and a metabolite. Red represents a positive correlation, while blue represents a negative correlation. *p < 0.05, **p < 0.01.

Based on the above detection of microbial taxa significantly associated with different grazing simulation treatments, we next conducted Spearman’s correlation analysis to determine whether and which yak rumen microbes shared an association with significant VFAs (Figure 5). This analysis indicated that the relative abundance of *p.251.o5* was negatively correlated with acetate and propionate concentrations (|*r*| > 0.5, *p* < 0.05); *Lachnospiraceae_ND3007_group* showed a positive correlation with valerate and isovalerate concentrations (|*r*| > 0.5, *p* < 0.05), and *Christensenellaceae_R-7_group* was negatively correlated with butyrate, valerate and isovalerate concentrations (|*r*| > 0.5, *p* < 0.05). In addition, the Ruminococcaceae_*NK4A214_group* bacterial ASV was significantly negatively correlated with all VFAs except isovalerate (|*r*| > 0.5, *p* < 0.05). Among fungal ASVs, the relative abundance of *Orpinomyces* was significantly positively correlated with acetate concentration (*r* = 0.43, *p* < 0.05), while *Piromyces* was negatively correlated with acetate and isobutyrate concentrations (*r* = −0.54 and −0.51, *p* < 0.05). At the genus level, *Penicillium* and *Candida* were positively correlated with valerate and acetate concentrations, respectively (|*r*| > 0.5, *p* < 0.05).

**Figure 6:**
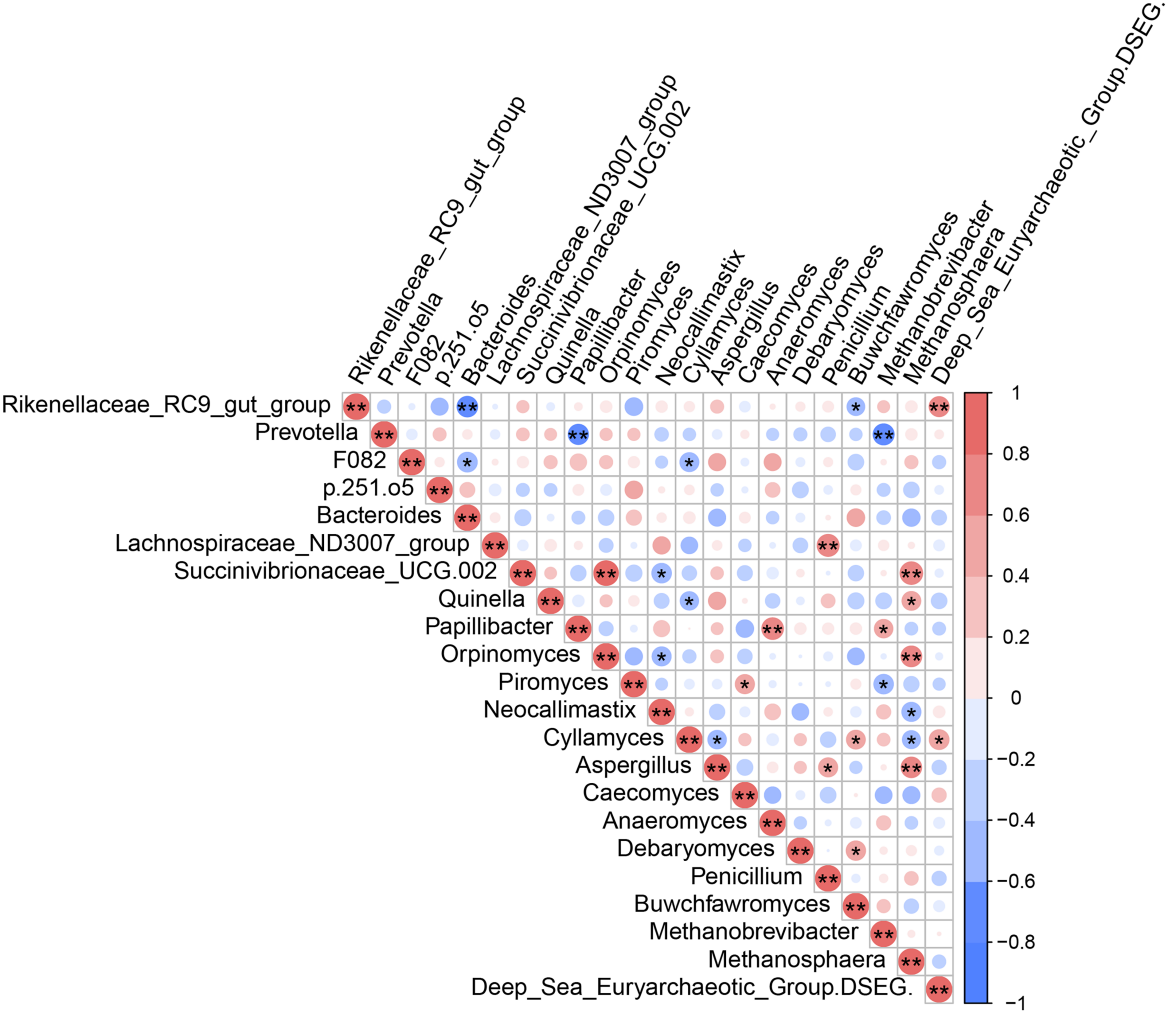
Mutual-correlation among the higher abundant genera of rumen microbial. Red color represents a positive correlation, while blue represents a negative correlation. *p < 0.05, **p < 0.01.

Analysis of mutual correlations among high-abundance rumen microbiota (bacteria, fungi and archaea) at the genus level (Figure 6) showed that *Deep_Sea_Euryarchaeotic_Group (DSEG)* was positively correlated with *Rikenellaceae_RC9_gut_group* (*r* = 0.60, *p* < 0.05) and *Cyllamyces* (*r* = 0.59, *p* < 0.05). *Methanosphaera* ASV counts were positively correlated with *Succinivibrionaceae_UCG-002*, *Quinella*, *Orpinomyces,* and *Aspergillus* (|*r*| > 0.5, *p* < 0.05); *Methanosphaera* was negatively correlated with *Neocallimastix* and *Cyllamyces* (|*r*| > 0.5, *p* < 0.05). *Rikenellaceae_RC9_gut_group*, *Prevotella,* and *F082* were negatively correlated with *Bacteroides* and *Buwchfawromyces*, *Papillibacter* and *Methanobrevibacter*, *Bacteroides,* and *Cyllamyces,* respectively (|*r*| > 0.5, *p* < 0.05). There were also significant positive correlations between *Lachnospiraceae_ND3007_group* and *Penicillium*, between *Orpinomyces* and *Succinivibrionaceae_UCG-002*, between *Papillibacter* and *Anaeromyces*, and between *Cyllamyces* and *Buwchfawromyces.* By contrast, negative correlations in ASV counts were identified between *Piromyces* and *Methanobrevibacter*, between *Cyllamyces* and *Aspergillus*, between *Succinivibrionaceae_UCG-002* and *Neocallimastix*, and between *Quinella* and *Cyllamyces* (|*r*| > 0.5, *p* < 0.05).

## Discussion

Yak is the only ruminant on the Qinghai Tibetan plateau that utilizes pasture year-round. Previous work has shown that yak rumen microbiota is robust to seasonal changes in forage availability, potentially enhancing the efficiency of fiber degradation during winter months (Huang et al., 2022). However, as important-fiber degrading microbes, the role of rumen fungi remains poorly understood in yak. In addition, environmental variability can strongly impact grazing studies (Wu et al., 2020), and current reports do not well-explain the role of rumen microbiota in the metabolic response to season-dependent changes in nutrient availability. Here, we explore the VFA profiles of rumen under simulated seasonal diet regiments, and characterize changes in rumen microbiota related to these seasonal diets by excluding environmental factors. Unlike grazing yaks, captive ruminants are subjected to less influence from complex seasonal shifts in environmental factors, and thus provide an ideal model for examining the metabolic effects of microbiota in high-altitude ruminants.

Volatile fatty acids reportedly participate in maintaining rumen homeostasis by providing an ideal acidic environment for the rumen microbiota (Bensadoun et al., 1962), and VFAs in rumen can provide approximately 75–80% of a host ruminant’s energy requirements (Lane and Jesse, 1997). Yaks have been shown to exhibit high VFA production in rumen but relatively low methane emission compared to cattle, possibly due to enrichment with energy metabolism-related genes, such as those involved in VFA production (Zhang et al., 2016). Ghimire et al. (2017) have reported various VFA profiles with different feed types. In the present study, rumen acetate, propionate, and total VFAs were higher in the GSG rumen (grazing simulation group, mimicking warm-season pasture) than in the SG (Supplementation group, mimicking winter grazing supplemented with concentrate) and NSG (nutrient stress group, mimicking winter grazing) rumen, possibly due to the availability of sufficient nutrients accompanied by a relatively high fiber/protein ratio (Liu et al., 2019). Previous work by Liu et al. (2019) showed that the forage group had higher acetate content and total VFA content than the supplemented group.

VFA production is closely related to rumen microbiota composition and diversity. *R. flavefaciens* and *F. succinogenes* are both known to produce large amounts of succinate through fiber degradation, which is increased by conversion to propionate (Fondevila and Dehority, 1996). *Prevotella* degrades starch and protein, producing propionate, succinate and acetate, which in turn affects VFA production (Strobel, 1992). Microorganisms can utilize acetate to produce butyrate *via* acetyl-CoA transferase and/or butyryl-CoA transferase (Hackmann and Firkins, 2015). In this study, we found that *p.251.o5* was negatively correlated with acetate and propionate concentrations. Among fungal ASVs, *Orpinomyces* which was at the higher level in GSG was significantly positively correlated with acetate and propionate content, further suggesting that VFA-related microbes are enriched in GSG, leading to functional differences in acetate metabolism in rumen. Besides, acetate and propionate account for a large proportion of total VFA, and their enrichment was aligned with an increase in total VFA content. *Piromyces* was significantly negatively correlated with isobutyrate concentration, which has been previously reported to affect the growth of rumen fungi (Elliott et al., 1987), further supporting the effects of VFAs in determining microbial community structure.

Rumen microbiota can cooperatively degrade polysaccharides, starch, and fibers in the feed, producing VFAs that provide energy for the host, and play a crucial role in host growth and development. In this study, we found that the *Bacteroidetes* and *Firmicutes* were the dominant phyla among the three treatment groups, which is consistent with previous studies (Xue et al., 2016; Hu et al., 2021; Jiang et al., 2021; Liu et al., 2021). *Bacteroidetes* can degrade soluble polysaccharides and starch in the rumen to produce acetate, propionate and butyrate, which can be utilized by the host (Rosewarne et al., 2014; Ahmad et al., 2020). Belanche et al. (2019) showed that a grazing diet increases the abundance of *Actinobacteriota*, which aligns well with our finding of enrichment for *Actinobacteriota* in the GSG rumen. By contrast, *Firmicutes* were found in higher abundance in the NSG than in GSG rumen, suggesting a possible role in facilitating host survival during nutrient deficiency. Similarly in fungi, *Neocallimastix* were more abundant in NSG and SG samples, possibly due to their reported functions in cellulose utilization (Wang et al., 2011; Boots et al., 2013) and protease production for protein degradation (Michel et al., 1993). Hernandez et al. (2019) found that *Cyllamyces* also participated in fiber degradation, which was abundant in NSG samples, while well-known fiber degrading bacteria were less abundant under nutrient stress.

Other than host influence (Henderson et al., 2013; Hu et al., 2021), feed type strongly affects microbial diversity and species richness (Henderson et al., 2015; Cremonesi et al., 2018; Wang et al., 2020). In the present study, bacterial ASV richness was higher in GSG samples than in SG samples, further supporting that a concentrated diet can reduce bacterial diversity (McCann et al., 2014; Ku et al., 2021), while fungal richness and diversity were higher in the NSG and SG rumen. As cellulose and hemicellulose-degrading bacteria (Zened et al., 2013; Sha et al., 2020; Cui et al., 2022), *Succinivibrionaceae_UCG-002* and *Rikenellaceae_RC9_gut_group* were enriched in GSG rumen, which had an intermediate fiber content (NSG > GSG > SG). At the species level, *R. albus* was more abundant in GSG rumen than that in SG rumen, while *F. succinogenes*, *B. fibrisolvens,* and *R. flavefaciens* were more abundant in SG samples than NSG. Together with the higher *Firmicutes* levels in NSG than GSG, enrichment with these known fiber-degrading bacteria (Forsberg et al., 1997) in GSG and even SG rumen, but their depletion in NSG samples, implies that nutrient deficiency can inhibit proliferation of known rumen taxa, while selecting for nutrient stress-tolerant, fiber-degrading bacteria and fungi.

Archaea account for ~2–4% of rumen microbes (Paul et al., 2015; Maman et al., 2020). Previous work by Liu et al. (2019) showed that archaeal rumen flora is affected by host factors and diet (Liu et al., 2019). In this work, *Euryarchaeota* was the dominant phylum, while *Methanobrevibacter* was the dominant genus, followed by *Methanosphaera*, which is consistent with other studies (Cunha et al., 2011; Sirohi et al., 2013). Compared to *Methanobrevibacter*, *Methanosphaera* spp. are remarkably enriched in “low hydrogen/methane” producing ruminants (Hoedt et al., 2018), further suggesting that hydrogen consumption by methanogens was decreased in GSG rumen, potentially enhancing VFA production.

PICRUSt analysis can be used to predict the metabolic function of bacterial communities. In the present study, superpathway of 5-aminoimidazole ribonucleotide biosynthesis was significantly enriched in GSG samples. 5-Aminoimidazole ribonucleotide was reported as a key intermediate in the biosynthesis of purine nucleotides and thiamine, as well as an important pathway in many cellular processes, such as cellular signaling and energy metabolism (Senecoff et al., 1996; Patterson et al., 1999). In addition, FUNGuild functional prediction analysis showed that the Plant_Pathogen, Endophyte−Plant_Pathogen, and Animal_Pathogen−Endophyte−Plant_Pathogen−Wood_Saprotroph were enriched in SG samples and indicated fluctuation response of the function of microbial communities among the different groups. Future validation towards understanding the metabolic function of microbial communities will require the integration of multi-omics analyses to identify the actual microbial and metabolic function response to various nutrient availability.

Yak rumen contain a complex network of symbiotic microorganisms that can synergistically ferment plant fibers, providing nutrients for themselves and the host (Ishaq and Wright, 2012). In this study, we identified interactions within and between rumen bacteria, fungi and archaea. Among fungi, *Cyllamyces* was negatively correlated with *Aspergillus*, while *Orpinomyces* was negatively correlated with *Neocallimastix*, suggesting that some rumen fungi may compete with each other. In addition, *Penicillium* (fungi) was significantly positively correlated with *Lachnospiraceae_ND3007_group* (Firmicutes), although further experiments and functional analysis based on metagenomic study are necessary to determine whether these clades can directly cooperate to utilize different components of the plant fiber.

This study comprehensively explores the response of yak rumen microbiota to simulated modes of seasonal feeding to exclude the impact of environmental factors. Our results show that different microbes in yak rumen gain dominance under (simulated) seasonal changes in nutrient availability, with relatively high nutrient availability promoting enrichment for known fiber degrading bacteria, and nutrient stress selecting for bacteria and fungi that are competitive under low nutrient availability and enhance the efficiency of host nutrient utilization. This study expands the scope of our understanding of microbial alteration and interactions under fluctuating nutrient conditions. Our findings provide a framework for future studies examining precise nutrient interventions or cold season probiotic treatments to enhance yak rumen function in nutrient utilization.

## Data availability statement

The datasets presented in this study can be found in online repositories. The names of the repository/repositories and accession number(s) can be found at: NCBI SRA database with accession number PRJNA863131.

## Author contributions

XH and RL conceived the study and designed the experiments. XY and XF performed DNA extraction and drafted the manuscript. XY, HJ, QZ, Basangwangdui, SD, and QZ coordinated in animal experiment and sample collection. XY, XF, and XH analyzed the data and contributed to data interpretation. All authors contributed to the article and approved the submitted version.

## Funding

This work was supported by National Key R&D Program of China (2021YFD1200904), Special Item of Regional Collaborative Innovation in Tibet Autonomous Region (QYXTZXLS2021-01, XZ202101ZD002N, QYXTZX-NQ2022-01, and QYXTZX-RKZ2020-04) and the Open Project Program of State Key Laboratory of Barley and Yak Germplasm Resources and Genetic Improvement (XZNKY-2021-C-014-K06) and National Modern Agricultural Technology System (CARS-37).

## Conflict of interest

The authors declare that the research was conducted in the absence of any commercial or financial relationships that could be construed as a potential conflict of interest.

## Publisher’s note

All claims expressed in this article are solely those of the authors and do not necessarily represent those of their affiliated organizations, or those of the publisher, the editors and the reviewers. Any product that may be evaluated in this article, or claim that may be made by its manufacturer, is not guaranteed or endorsed by the publisher.

## Footnotes

^1^ https://www.arb-silva.de/

^2^ https://unite.ut.ee/

^3^ https://huttenhower.sph.harvard.edu/galaxy/root

^4^ https://www.ncbi.nlm.nih.gov/sra/?term=PRJNA863131

## Supplementary material

SUPPLEMENTARY FIGURE S1 Amplicon sequence variants (ASV) of rumen microbiota in GSG, NSG and SG. (A) Bacteria. (B) Fungi. (C)Archaea.

SUPPLEMENTARY FIGURE S2 Classification of the rumen microbial composition at the phylum level across the different nutrient simulations in different grazing patterns. (A) Bacteria. (B) Fungi. (C) Archaea. (D) Bacterial phylum with significant changes under various nutrient simulations. (E) Fungal phylum with significant changes under various nutrient simulations. Asterisks indicate significant difference between the three groups (**P* ≤ 0.05; ***P* ≤ 0.01).

SUPPLEMENTARY FIGURE S3 Linear discriminant analysis (LDA) of rumen microbial communities. (A) Fungi. (B) Archaea. (LDA cut--off of +/−2.0).

SUPPLEMENTARY FIGURE S4 Bacterial, archaea and fungal function profiles of GSG, NSG, and SG by KEGG pathway database and the FUNGuild database. Heatmap plot showing significant differences among the three groups. (A) Bacteria. (B) Fungi. (C) Archaea. * *P* < 0.05, * * *P* < 0.01.
