# Supplementary figures and images for: Simulated seasonal diets alter yak rumen microbiota structure and metabolic function
Source: Front Microbiol. 2022 Sep 23;13:1006285. doi: 10.3389/fmicb.2022.1006285 (PMC9538157; doi:10.3389/fmicb.2022.1006285)

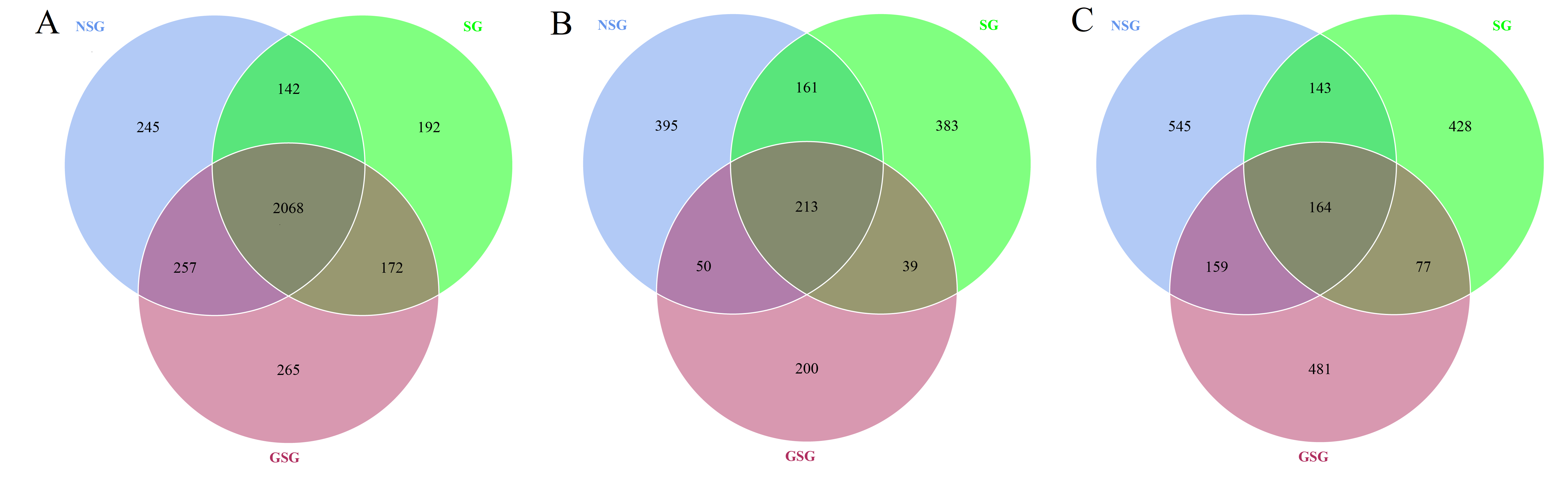

Supplement: SUPPLEMENTARY FIGURE S1 — Amplicon sequence variants (ASV) of rumen microbiota in GSG, NSG and SG. (A) Bacteria. (B) Fungi. (C)Archaea. [file Image_1.TIF]

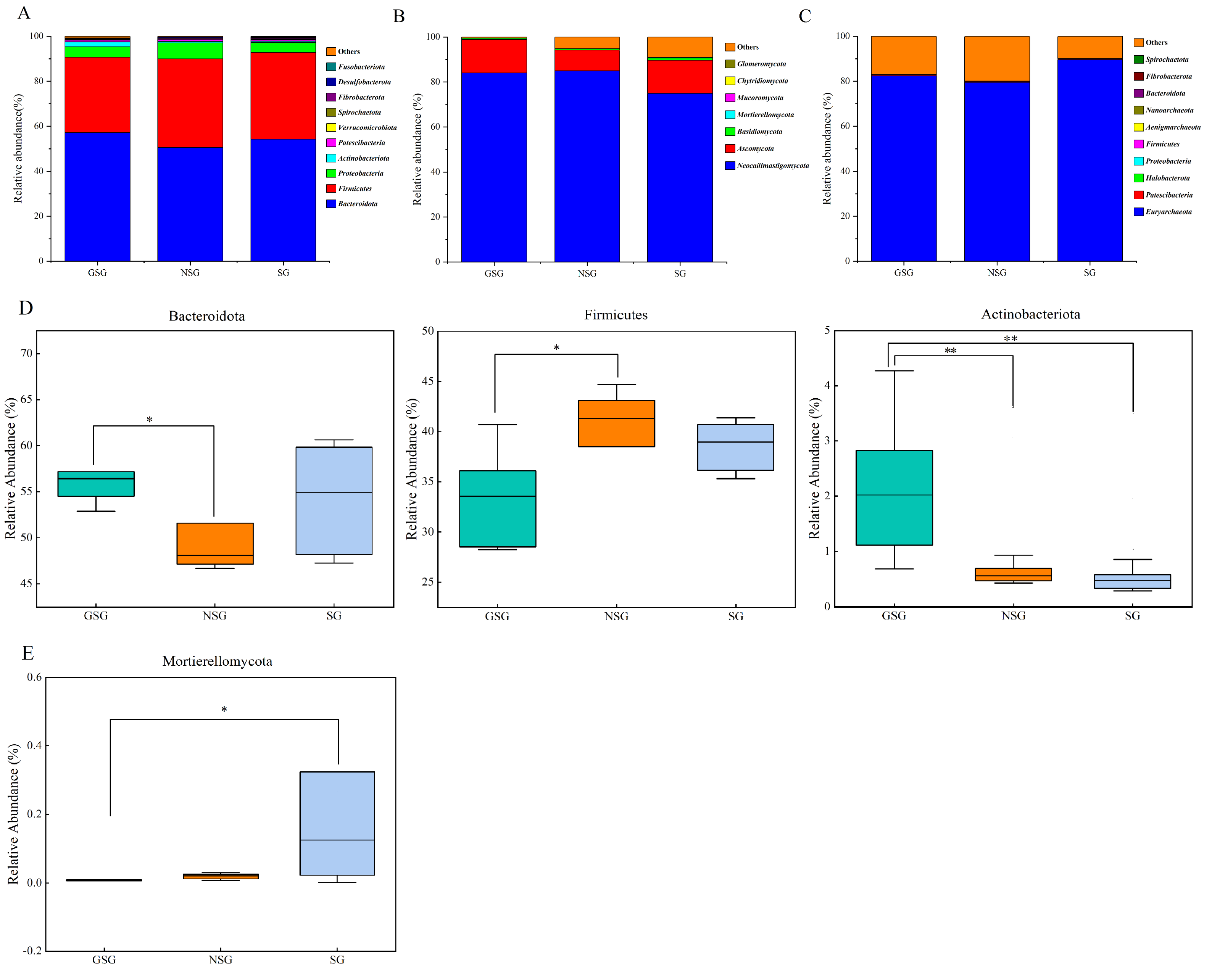

Supplement: SUPPLEMENTARY FIGURE S2 — Classification of the rumen microbial composition at the phylum level across the different nutrient simulations in different grazing patterns. (A) Bacteria. (B) Fungi. (C) Archaea. (D) Bacterial phylum with significant changes under various nutrient simulations. (E) Fungal phylum with significant changes under various nutrient simulations. Asterisks indicate significant difference between the three groups (*P ≤ 0.05; **P ≤ 0.01). [file Image_2.TIF]

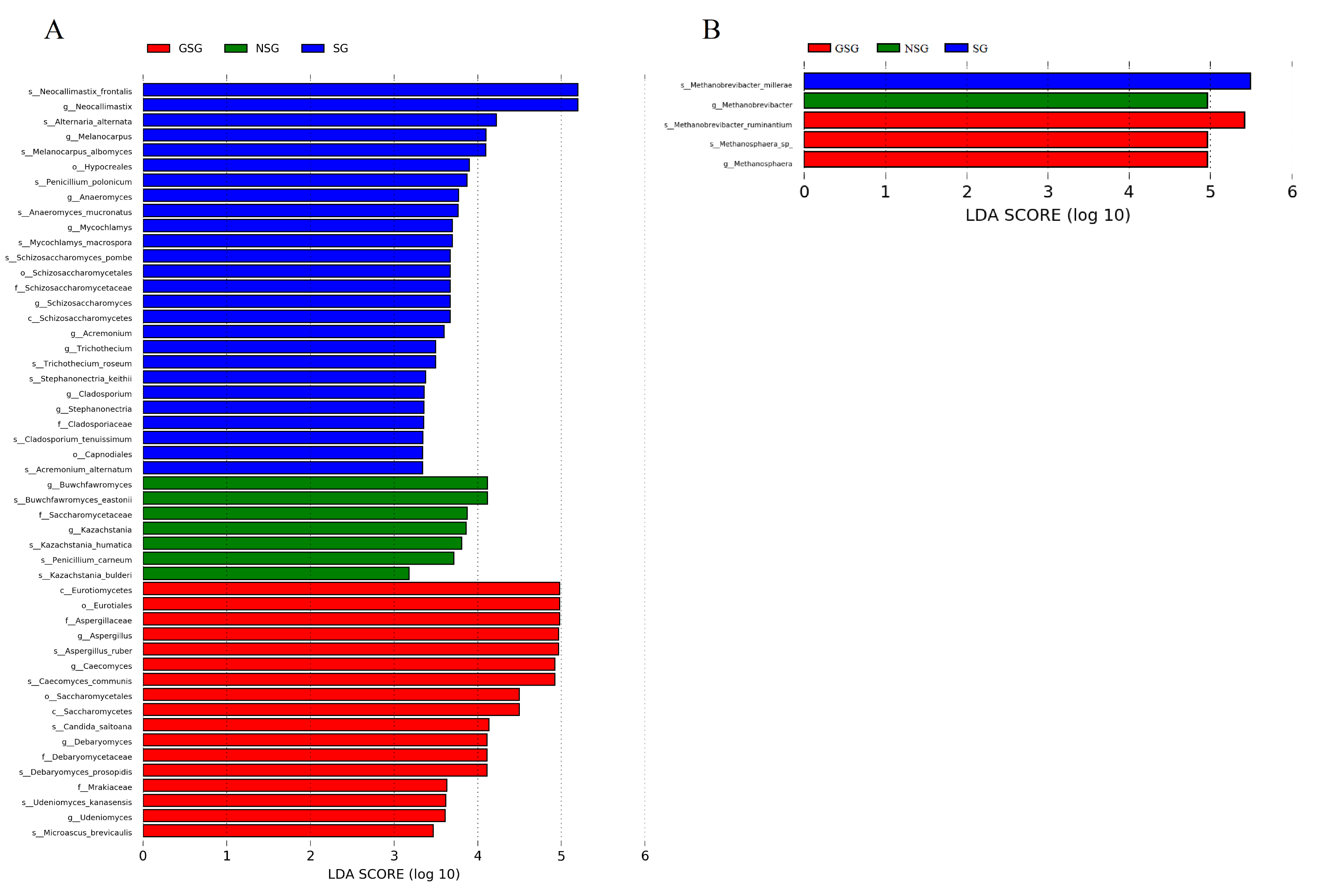

Supplement: SUPPLEMENTARY FIGURE S3 — Linear discriminant analysis (LDA) of rumen microbial communities. (A) Fungi. (B) Archaea. (LDA cut--off of +/−2.0). [file Image_3.TIF]

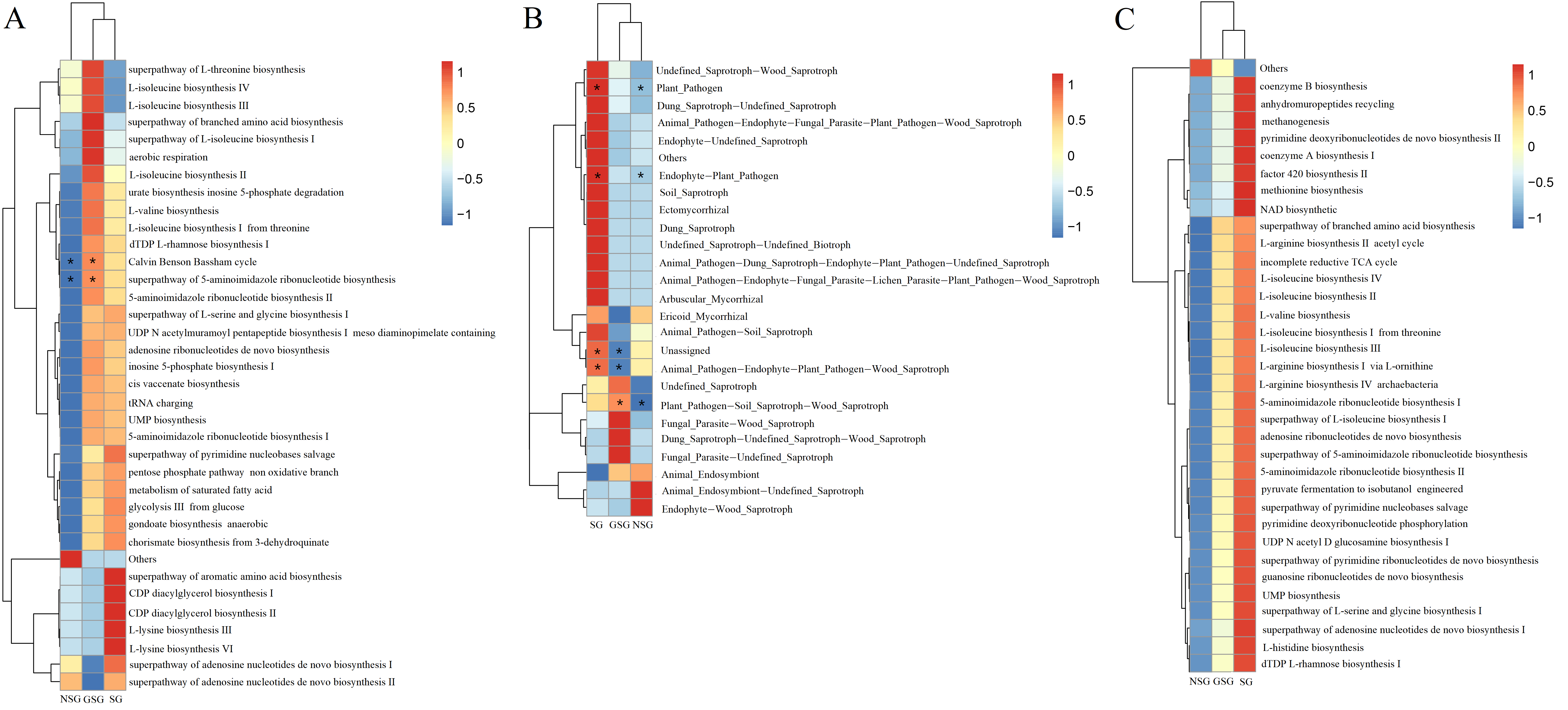

Supplement: SUPPLEMENTARY FIGURE S4 — Bacterial, archaea and fungal function profiles of GSG, NSG, and SG by KEGG pathway database and the FUNGuild database. Heatmap plot showing significant differences among the three groups. (A) Bacteria. (B) Fungi. (C) Archaea. * P < 0.05, * * P < 0.01. [file Image_4.TIF]
